# Community-dwelling older adults’ needs and acceptance regarding the use of robot technology to assist with daily living performance

**DOI:** 10.1186/s12877-019-1227-7

**Published:** 2019-08-05

**Authors:** Yeon-Hwan Park, Hee Kyung Chang, Min Hye Lee, Seong Hyeon Lee

**Affiliations:** 1 College of Nursing of Seoul National University, 103, Daehak-ro, Jongno-gu, Seoul, Republic of Korea 03080; 2 Research Institute of Nursing Science of Seoul National University, 103, Daehak-ro, Jongno-gu, Seoul, Republic of Korea 03080; 3 College of Nursing Senior Health Research Center of the Health & Science Institute, Gyeongsang National University, 15, Jinju-daero 816beon-gil, Jinju-si, Gyeongsangnam-do Republic of Korea 52727

**Keywords:** Ageing, Independent elderly, Mixed-method, Perception, Robot technology

## Abstract

**Background:**

The rate of aging in Korea is extremely fast compared to major countries. We examined the key demands of community-dwelling older adults with regard to Connected Active Space technology, which provides tailored assistance with daily living performance through robotic services.

**Methods:**

This study is based on a mixed-method design, through a quantitative survey (*n* = 234) first phase, followed by a qualitative study with focus group interviews (n = 23) to explore the needs and acceptance of community-dwelling aged people concerning the application of robot technology in their daily lives.

**Results:**

The scores concerning the need for and acceptance of robot services to assist daily living performance were high, at 7.2 and 7.9 out of 10 points, respectively. Further, for both needs and acceptance, timely reaction to emergency situations, early detection of emergency situations, help to locate objects, assistance with mobility, and assistance in memory recall were prioritized (in that order). In a thematic analysis of qualitative data from three focus-group interviews, a ‘mismatch between desires and functional capacity’ was the core characteristic of living as an older person and ‘being a friend and helper’ was the most desired trait of a robot service.

**Conclusion:**

Although most of the participants lived independently, they regularly experienced difficulties regarding buying products, transportation, using phones, and preparing meals. If appropriate assistance technology is developed, this population can maintain its independence. Thus, it is necessary to address main needs, including detecting and addressing emergency situations, locating objects, assisting mobility and memory recall, and assisting with daily living performance. New robot services that can be tailored to the functions or abilities of the elderly must be developed based on individually collected information.

## Background

In Korea, population aging is accelerating at an unprecedented rate. In 2020, when the baby boom generation becomes an aged population, seniors are expected to account for 15.6% of the total population, which would constitute an aged society, and by 2026, this is set to reach 20.8%, creating a post-aged society [1]. Despite this rapid increase, the life expectancy of the Korean elderly, excepting the periods during which they suffer from a serious disease (or their “health-adjusted life expectancy”), is relatively low. As the number of elderly people requiring care rises, the care-giving burden also grows. In 2004, there were 8.2 members of the working population available to care for each senior citizen; however, by 2030, this ratio will have fallen to 2.8:1 [2]. Thus, it seems that the care-giving burden is becoming more severe, not just for families, but also for society in general. In particular, this burden is increasing the risks for senior citizens who live alone, in terms of their safety and their ability to maintain their lifestyles. The prevalence rate of chronic diseases among the elderly, which reflects their health conditions, is 88.5%, and when daily living assistance is considered, dependent seniors account for 49.7% of the total senior population [1].

It is critically important to mitigate the effects of aging, improve older adult’s life, and improve overall quality of health environment. Smart aging addressed those challenges by intelligently utilizing modern biomedical, digital healthcare, computing, and communication technologies [3]. Previous literature shows that smart aging promotes the well-being of aging population by seamless integration of information technology, medical systems and devices, bio-technology, and robotics [3, 4]. Considering this, it is necessary to identify the characteristics and needs of the elderly as service consumers and to use these to develop advanced service technologies that support elderly people’s daily living in a manner that conforms with their various needs and to both their health and their illnesses.

Various studies have advocated the adoption of rapidly-advancing technologies for supporting elderly people’s lives (the Fourth Industrial Revolution and AI, to name a few) [5]. However, before advancing such technologies or products, it is necessary to identify the elderly population’s perception and acceptance of such technologies, as identifying this population’s key needs are essential for setting the direction of product development when introducing such information-communication-technology (ICT)-based robotics. However, in spite of the promising future heralded by the advancement of the Fourth Industrial Revolution and AI, research on the perceptions of the seniors who will actually live with such robots and use such technology in their daily lives remains scarce. Recently, overseas researchers have reported on elderly people’s attitudes towards robot services and their technological acceptance and experience of using them by studying elderly people in nursing homes [6], geriatric rehabilitation centers [7], and in their own homes [8–11]. However, Korea-based studies remain insufficient. In particular, the development of robots for care for older people is accelerating ahead of our entry into a post-aged society in early 2026, and the number of applications for elderly care robots grew at an average rate of 72 per year from 2016 to 2018 [12]; little is known about the perception of older people’s own needs or their acceptance of robot technology in Korea.

In order to develop effective robot technology services, a more accurate understanding of elderly people’s health conditions and characteristics is needed. This is because identifying elderly people’s technological acceptance and consumer needs regarding the application of ICT robot technology in their daily lives is necessary in order to set the direction of product development. In particular, it is necessary to identify through qualitative research that which they perceive as the essential elements for supporting their lives. Therefore, it is imperative to conduct research that offers descriptions and structures concerning community-dwelling elderly people’s needs and acceptance of robot services.

This study aims to identify this population’s needs and acceptance of robot services that have the potential to address their self-perceived needs and deficiencies, and to provide additional insight into the responses to the survey through the use of focus group interviews with community-dwelling elderly people who live independently.

## Methods

### Study design

**Fig. 1:**
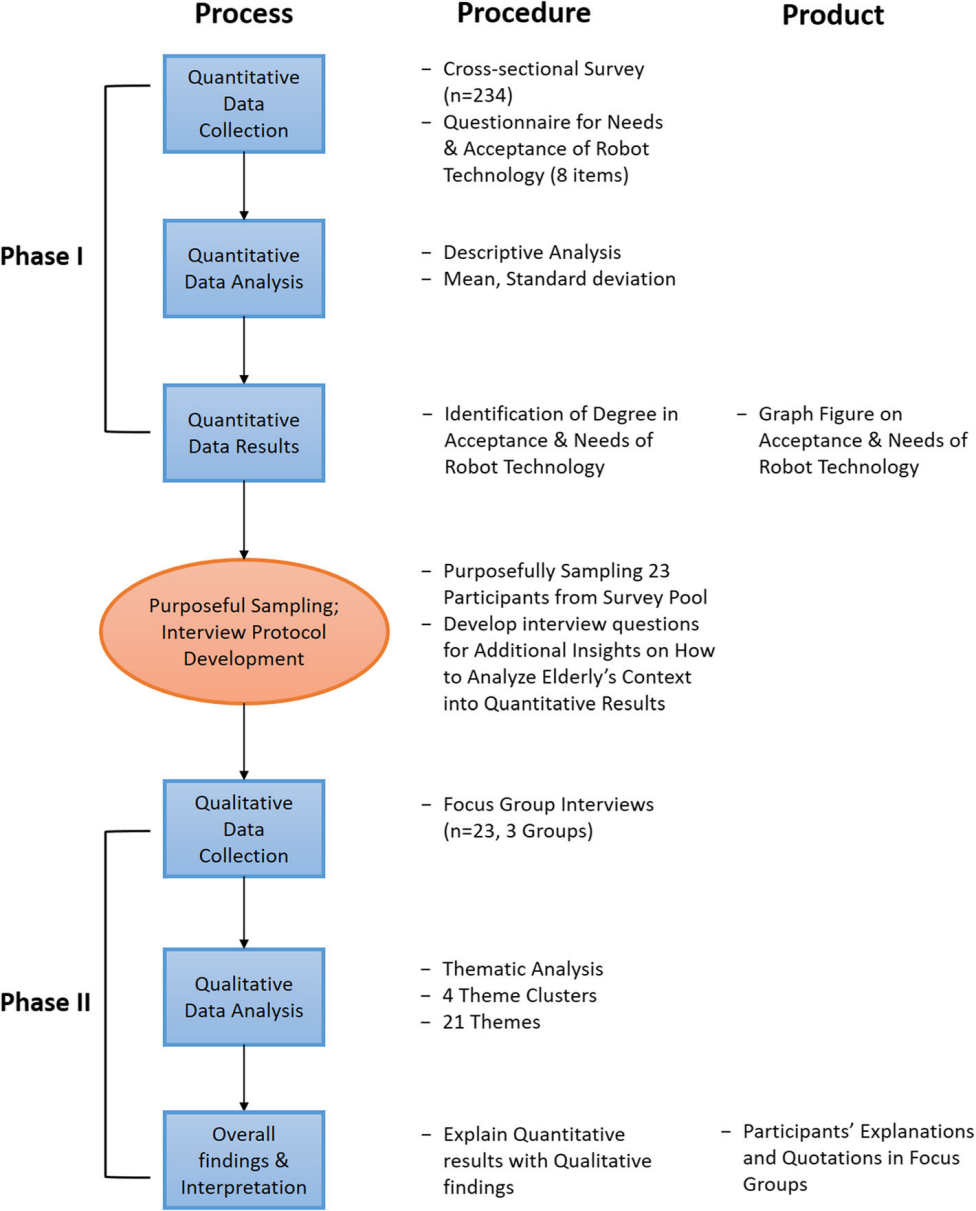
Process flow diagram of the procedures for this sequential explanatory mixed methods study

To explore the acceptance and needs of robot technology in community-dwelling older adults, we used what Creswell calls a “sequential explanatory mixed methods design” [13, 14] to explain the context of the quantitative analysis results from participants’ voices for additional insights (Fig. 1). In the first stage, quantitative findings were obtained from analysis of cross-sectional survey (*n* = 234). In the second study phase, we used focus group interviews (*n* = 23) to help explain the quantitative results.

### Participants’ recruitment

The subjects of this research were selected through convenient sampling. The recruitment of participants took place in two phases. In the recruitment of participants, the aim was to have a broad, representative spectrum of elderly in Korea. The selection of participants and organization was based on the advice of healthcare opinion leaders and the authors’ knowledge about the field.

### Ethical considerations

The participants from the survey and interview study were informed of the study’s purpose, mode of participation and confidentiality. All participants reported that they understood the objective and method of this research and voluntarily signed consent forms. Participation was entirely voluntary, and data were handled confidentially. All study procedure was approved by the Seoul National University Institutional Review Board (Approval No. 1608/001–001), and all participants underwent a written informed consent process.

### Quantitative data collection and analysis (phase I)

In the first phage, the subjects of the quantitative data analysis were elderly individuals aged 65 or over, all of whom were sourced from one of the senior welfare centre in J district, Seoul in Korea. The quantitative data were collected over four days, from August 1 to 4, 2016, through surveys answered by 234 senior citizens.

To develop this self-reported needs and acceptance tool, the researchers followed a systematic process for constructing survey tools [15], particularly that of reviewing the literature, developing or adapting tool items, constructing the tool, and pilot-testing the draft tool. Preliminary items were developed based on the information from the Center for Intelligent & Interactive Robotics at the Korea Institute of Science and Technology (KIST) as an initial guide. In addition to their advice, relevant literature was searched on ICT, gerontechnology, and smart aging, and healthy aging by the authors. The preliminary tool was reviewed and piloted with three faculty members at KIST and three gerontological nursing faculty. Two rounds of iterations were undertaken to refine the content and wording of items in the survey measurement. The resulting 8-item tool asked respondents to rate their acceptance of and needs for Robot technology.

Connected Active Space (CAS) needs were represented by eight categories, including early detection of emergency situations and reacting to emergency situations in time, recording food intake, locating objects, assisting with mobility, recording and recalling memories, and logging daily activities. These were scored using the Visual Analogue Scale (VAS), with responses ranging from “never need” (zero points) to “very much need” (10 points). CAS acceptance was also determined using the same eight categories and was also scored using the VAS, ranging from “never accept” (zero points) to “very much accept” (10 points).

All analyses were conducted with SPSS software (Statistical Package for the Social Sciences v. 21.0). The general features of the subjects and their acceptance of and needs for of robotic technology were analysed based on descriptive statistics, application frequency, percentage, means, and standard deviation.

### Qualitative data collection (phase II)

For qualitative interviews for this study, purposive method was used to sample participants from the survey phase. The intention was to obtain a knowledgeable perspective on older adults’ perception about robot technology. The goal of this type of qualitative research is not the generalizability or representativeness of the study findings, but rather providing additional insight on the responses to the survey as well as understanding the context relate factors of their acceptance of and needs for robot technology services with older adults’ desire and dread in their daily lives.

Before conducting the focus group interviews, the authors considered the overall goals, clarified the main theme of the research, and created questions for the interviews [16, 17]; this allowed them, through qualitative data collection, to obtain information regarding the participants’ perceptions and any experiences relevant to their needs for robot services in their daily lives.

In the second phase, qualitative data were collected through three focus group interview conducted from 22 to 26 August, 2016; 23 older adults involved in the first phase, with six to nine individuals in each group. These latter individuals were recommended by the centres’ nurses or voluntarily agreed to participate in the interviews, as they were able to engage in active communication and answer abstract questions, such as items concerning life as an older person and impressions of robot technology.

The interview protocol consisted of three parts. The introduction had open-ended questions about what living as an older person was like. Then we showed them three video clips that described an old man living with a humanoid robot named Pepper that provides assistance in the home, an old woman suffering from cognitive disorders who was living with Paro, an emotional therapeutic robot in the form of an animal, and a middle-aged patient with a stroke who wears an exoskeleton device for climbing stairs. These three examples of robots were chosen based on the research of Broekens, Heerink, and Rosendal [18]. The authors differentiated between robots in aged care that have a purely assistive function, as in smart wheelchairs or exoskeletons, and those that have a communicative function, in the sense of providing information to their customers and interacting with them. Social robots can be divided into two groups: companion-type robots and service-type robots. According to the classification of the robots presented in the above literature, we presented Paro to older adults as a kind of companion-type robot, Pepper as a kind of service-type robot, and the exoskeleton robot as a purely assistive robot that helps people climb stairs. Followed by questions asking, ‘With which aspect of your daily life do you most require another person’s assistance?’, ‘If there was a robot capable of assisting you in your daily life, what aspect would you prioritize?’, ‘How do you think a robot can help you in regard to your most pressing needs?’, and ‘How can a robot help keep your biggest fear from coming to pass?’ As transition topics, we asked questions about their desires and concerns as older adults.

The interviews were conducted in such a way that each participant answered when the moderator asked key questions, and their answers were recorded immediately. All of the interviews were recorded, and the interviewees’ observations, thoughts, and feelings were noted and used for analysis.

### Focus group data analysis (phase II)

The collected data consisted of the manuscripts extracted by the focus groups, notes that researchers handwrote during the interviews, and debriefing notes written after the focus group and personal interviews, producing approximately 112 A4 pages in total.

The authors held a total of twelve meetings to confirm the analysis processes, including topics such as deciding when to cease data collection, finding concepts, and defining categories. While doing so, they continuously compared the provisional categories with various similar situations, modifying and revising the list as appropriate.

The first analysis concerned open coding, in which each line of the recorded tape was converted into one unit, and words, sentences, and paragraphs from each analysis unit that were deemed relevant to the research objective were logged. Analysis units with similar content and aspects were categorized in the first analysis step, then, in the second analysis step, each category was structured with subcategories; further, by using comparative analysis for more interview materials, the authors added or edited categories. When reaching the point where no more new themes could be created, data collection was halted, and the main themes were specified using the obtained themes.

Adhering to the assessment items for qualitative research [19, 20], the authors ensured the rigor of this research and double-checked the participants’ understanding by summarizing and explaining the interviews immediately after finishing the interviews.

Moreover, the authors also checked whether the interviews were accurately recorded by randomly comparing the recorded tapes and voice-recording files. Next, the authors asked two male and two female seniors, who were not participants, to review the results and ensured the results’ applicability by checking that the results were suitable for elderly people.

Lastly, the authors left audit trails so that other researchers could track the entire process of taking notes, keeping a daily log, and performing analysis. The qualitative research team was in charge of deciding whether to continue data collection and leaving audit trails, reconfirming the analysis process, and creating the theory during the regular meetings, and also for maintaining consistency and neutrality. In addition, they shared the results with three research participants in order to verify their meaning, and when writing the research, they specifically described the choices and features of the participants and, in the research method, how they collected and analyzed the data.

## Results

The results consisted of quantitative data (collected from questionnaires that surveyed elderly people’s needs for and acceptance of robot services and what they desired from robots, in addition to their general characteristics) and qualitative data (where the authors analysed the self-perceived characteristics of being an old person and their needs concerning robotic technology services through focus group interviews).

### Survey sample characteristics and results (phase I)

For the 234 participants, the average age was 75.7 years (± 5.8 years, ranging from 65 to 96 years old), and 166 (70.9%) were female and 68 (29.1%) were male (Table 1). Their average level of education was 7.6 years (± 4.9), and 105 people (44.9%) reported living alone while 129 (55.1%) were living with their family or acquaintances.

**Table 1:** Summary of the Sample Characteristics

Participants	Survey in Phase I (*n* = 234)	Focus groups in Phase II (*n* = 23)
Sex (*n*, %)		
Female	166 (70.9)	20 (86.9)
Male	68 (29.1)	3 (13.1)
Age (M ± SD) (years) (range)	75.7 ± 5.8 (65~96)	75.5 ± 5.5 (66~85)
Education level (M ± SD) (years)	7.6 ± 4.9	8.9 ± 3.8
Living alone (*n*, %)	105 (44.9)	12 (52.2)
CAS needs score (0–10) (M ± SD)	6.1 ± 4.0	7.2 ± 1.8
CAS acceptance score (0–10) (M ± SD)	6.3 ± 3.9	7.9 ± 1.8
Number of robot functions required (0–6) (M ± SD)	2.7 ± 1.6	3.4 ± 1.5

Note. *CAS* connected active space, *M* mean, *SD* standard deviation

**Fig. 2:**
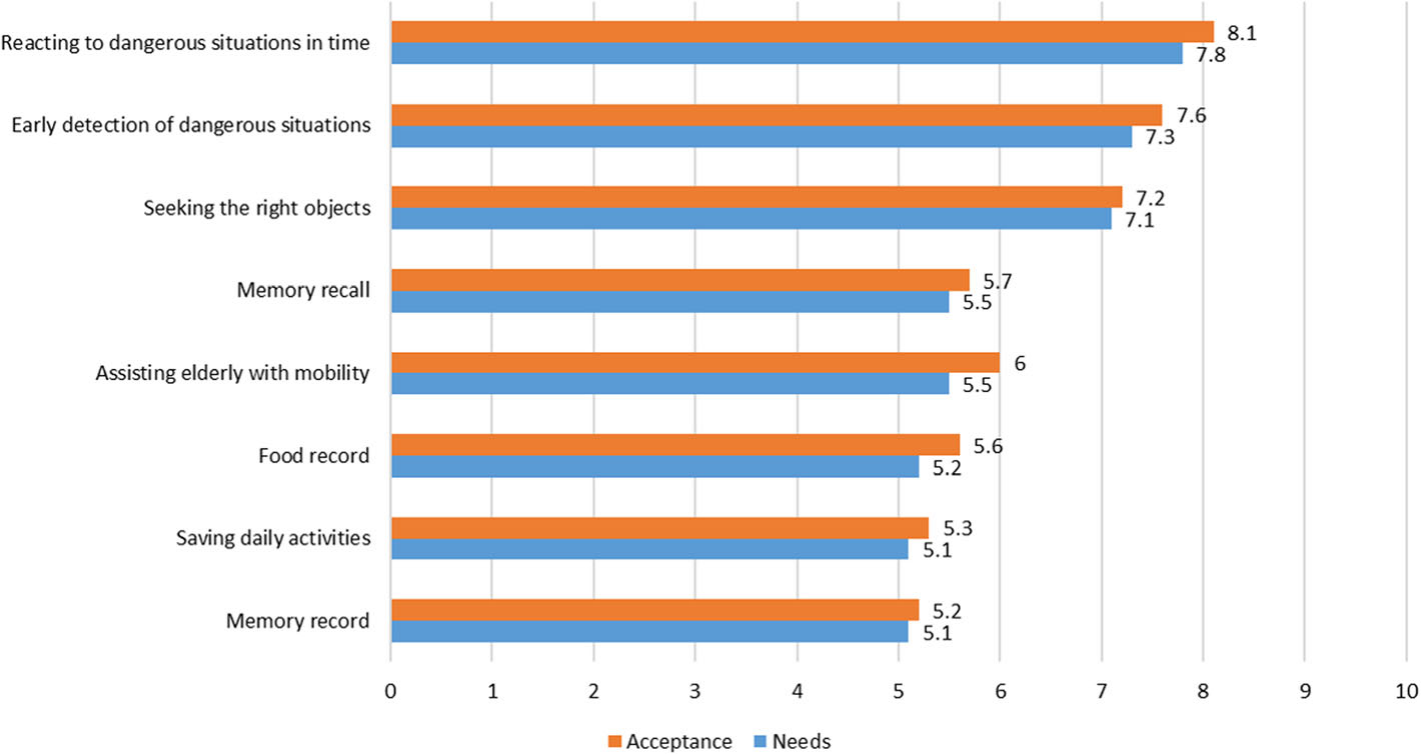
Mean Scores per Item on a Ten-point Scale, Indicating the Acceptance of and Needs for Robot Technology (*n* = 234)

Table 1 illustrates the results of the analysis of the quantitative data regarding the participants’ self-perceived characteristics and their needs concerning robotic technology services. Among the eight categories of CAS technologies, the average CAS needs score was 6.1 out of 10 points (± 4.0, 0~10) and the average CAS acceptance score was 6.3 (± 3.9, 0~10). The categories for which high needs scores were reported were, in order of score, reacting to emergency situations, early detection of emergency situations, locating objects, assisting with mobility, and memory recall, and the same answers were reported for CAS acceptance. Regarding the number of functions for which robot help was considered necessary, the respondents answered 2.7 (±1.6) on average, out of a total of five. Technology for providing information was chosen by 66.7%, while 83.8% said that they needed a robot to help detect emergency situations and call ambulances or hospitals (Fig. 2 and Table 1).

### Focus group interviews (phase II)

In Table 2 described four theme clusters that emerged in the second phase of this study according to a priori categories from the interview questions, which was conducted using the data sourced from the three group interviews. In this study, the classification standard for older persons and the necessary level and functions of robotic technology services for this population, as perceived by the participants, were grouped under the following five themes.

**Table 2:** Overview of Themes Identified through Focus Group Interviews

Category	Theme clusters	Themes
Characteristics of living as older persons	Mismatch between desires and functional capacity	Deteriorating abilities Fears Desires Composure Reputation
Desires as an older person	Living happily and dying quickly	Learning Interaction Dying well Pleasure Domestic environment Equability
Things older people dread	Losing dignity	Dying Offspring Sorrow Disease
Desired robot service	Being a friend and helper	Housework assistance Companion Teaching assistance Coping at Emergency Safeguard Physical assistance

#### Characteristics of living as an older person: Mismatch between desires and functional capacity

The authors used thematic analysis for the responses provided by the 23 participants of the focus group interviews and used this to determine the characteristics of living as an older person. The participants’ answers were deterioration, fear, hopes, composure, and decency, which converged on one theme, ‘a mismatch between desires and functional capacity.’ This means that even if they wanted to do something, like traveling or learning, they could not fully engage in the activity as a result of a gap between the abilities of their minds and bodies. The following was selected as the most relevant quotation in this regard.

First, I’m losing my memory, scared of everything, and feeling pains all over my body... (interruption)... I feel that my health is getting worse every year, my legs do not move as I want, and I feel tired... (interruption)... I cannot hear, taste, or sense very well. However, I still have the same desire to learn that I had when I was young. So, when I try to learn, I realize that I am now old, and this makes me sad. I have so many things I want to do in my mind, but my body cannot keep up with my mind (Female participant 11).

#### Desires as an older person: Living happily and dying quickly

Analysing source data for “what the elderly currently want to do” showed that the participants valued learning, interacting, dying well, having fun, a family environment, and peace. Considering this, the theme “living happily and dying quickly” was created, and the following was selected as a representative quotation, “I want to live happily, suffer a short illness, and die” (Female participant 7).

3) Things older people dread: Losing dignity.

In the source data for “things older people dread,” the participants said that they were afraid of death, worrying about their children, regret, and illness. Consequently, the relevant theme became “losing dignity after a long-term illness or dying alone,” represented by the following quotation: “what I’m most afraid of is that I may show an embarrassing side of myself to others” (Female participant 6).

#### Desired robot service: Being a friend and helper

When asked about a function that robots could perform on behalf of the participants or for other people, the participants emphasized that they desired robots to be like friends as well as helpers, robots that could assist with house chores, be a friend, and address physical inconveniences. The relevant quotations were “robots will be welcome if I can return to my home, where they can bring me water and live and play with me...” (Male participant 13) and “I really need robots who can help me climb stairs” (Female participant 21).

## Discussion

In this research, the needs for and acceptance of the elderly regarding robotic technology services were determined to be significantly high, which is consistent with the study Seelye et al. [20] conducted on elderly people who were able to live independent lives and had no cognitive disorders, similar to the participants of the present study; the researchers determined that they had a positive utility and acceptance of robotic technology services. However, this differs from the findings of a study conducted by Wu et al. [21], in which 11 seniors lived with robots for four weeks, after which their acceptance and level of use of the robotic technologies were analysed. It was consequently found that their acceptance was low and their opinions of the usefulness of the robots in their daily lives were also negative. Wu et al.’s finding is supported by Smarr et al. [22], which analysed 21 seniors’ openness to and preferences regarding robotic technology services; here, the participants showed low preference toward the introduction of robot services in their daily lives. These studies seem to have uncovered low levels of acceptance toward robotic technology services, not just because of the complexity of using robots, but also because of a feeling of stigma that may arise as a result of using robots to alleviate an impairment. We feel that the results of our study differed because the participants watched video clips and were exposed to research that presented a positive image of the application of such robots, without actually using them.

The participants in this study selected assistance, such as early detection of and responding to emergencies or helping people with cognitive disorders find appropriate objects, as the kind of robotic-based assistance they most desired in their daily lives. This is consistent with the results of a study conducted by Pino et al. [23], which identified elderly people’s preference boundaries regarding robot functions. It determined that this population most prefers support for their cognitive functions (38%). Further, in a Korea-based study, Oh et al. [24] showed that silver care robots contribute to reducing cognitive deterioration and improving daily living performance. However, the study also highlighted that the elderly engages in limited use of robots’ entertainment functions, and that they experience difficulty directly using and understanding robots. Therefore, just like the participants in this research, it seems that it is necessary to create personalized robots for community-dwelling elderly people who live independently; this was also suggested in Pino et al.’s study [23], where such personalization was identified as a priority. In this paper, since the elderly people wanted to live “self-directed lives” or “self-driven lives,” just as in the key theme shown in the focus group interviews, it would be necessary to create and offer services tailored to people’s needs and level of independence.

Seniors who can live independently experience a mismatch between their deteriorating memories and physical functions and their desire to continue living healthily and happily. Such a mismatch is also associated with the fact that they cannot obtain desired support from their offspring when facing emergency situations or safety issues they cannot address by themselves. Also, as they wish to learn and interact with others happily while living, and then to die in peace; they are afraid of losing their dignity as a result of a long-term illness and of dying alone. As the number of elderly people living alone in Korea has tripled from 540,000 in 2000 to 1.44 million in 2014 [1], in the future, many elderly couples may be unable to maintain their daily life standards, because, in many cases, both will have health issues; this means that caring for these people will create a great burden on the state. Further, geriatric diseases are mostly accompanied by other complex diseases, have ambiguous symptoms, change unexpectedly, and require comprehensive and constant care [25]. Thus, it is necessary to develop technologies that can detect abnormal physical conditions at an early stage, such as through robots’ monitoring functions. Such information can then be communicated to hospitals, and appropriate decisions (such as emergency responses, diagnosis, and treatment) can be made.

In the earlier stage of this study, the authors predicted that the elderly’s main needs regarding robotic technology services would relate to assistance. However, while analysing the interviews, the authors reached the conclusion that the participants sought to continue their “self-directed lives” regardless of their physical conditions and physical deterioration. This can be considered a theme reflecting self-regulation and self-control, which are the features of successful aging [26] and show the healthy stubbornness of the older generation to maintain their way of life despite living in an era of high dependency on cutting-edge technology. Therefore, at the marketing stage for robots, the elderly should not be regarded as dependent consumers who desperately need an “assistant” as a result of their deteriorating functions. Instead, companies should consider how robotic technology can be positively “applied” to the help realize the “desirable lives of the elderly.”

Previous surveys [27] also showed that older adults viewed socially assistive robots (SARs) as being capable of fulfilling variable and useful servant-like functions, providing a complete safety system (or at least a component of one), giving cognitive assistance, offering valuable sources of entertainment, and being linked to companionship. In Korea, social and family relationships have consistently remained at a certain level, but with the expectation that sons and daughters support their parents as they age having been reduced significantly. Among those in their 80s and above, internal diversity is also significant, as for many, their relationships with their offspring have weakened [1]. Therefore, social attention should be focused on people aged 70 or over and who have weak social and family relationships and policy interventions should be implemented accordingly. In other words, primary informal relationships, such as relationships with spouses or children, weaken as age increases. However, the frequency of seeing friends, neighbours, and acquaintances is not associated with age [1]. Considering this, to complement help from local communities, it is necessary to devise measures that strengthen robots’ integration into the family, which can result in enhanced social integration for the elderly.

For the preventive aspect, based on the results of this study, the elderly would be able to utilize robots’ monitoring and connecting functions in order to detect and respond to dietary imbalance issues at an early stage, which is particularly relevant for this population. In western countries, assistant technology is applied in elderly care centres on a massive scale. Further, in response to demographic changes, such as increases in the number of elderly people requiring treatment and care, technology that specializes in elderly care has been developed and applied [28, 29]. According to previous studies, robots should be able to capably fulfil the roles of a housekeeper and to assist with mobility.

Considering the results discussed so far, the community-dwelling elderly require robots who can ‘be a friend and helper’, meaning that they wish to maintain their daily lives and to concurrently interact with others. As elderly people who pursue a self-directed life are the main consumers of such robots, CAS-technology-based services should create functions and delivery methods that are tailored to the level of independence and needs that individual seniors expect.

## Conclusion

This study aimed, through surveys and focus group interviews, to analyse community-dwelling older person’s self-perceived needs and acceptance of robotic assistance in their daily lives as well as to identify the categories of this population’s desires of robots. It is hoped that the data obtained can serve as a basis for developing CAS-based robot functions that fulfil this population’s needs.

Based on our findings, it seems necessary to develop robotic technology services that are tailored to individual persons and that can adapt based on information collected as elderly people age. Considering this, it is necessary to develop assistant technologies that can prioritize elderly people’s needs that can also perform early detection of and execute responses to emergency situations, help individuals with cognitive issues locate appropriate objects, and assist in regard to mobility and memory recall.

Based on the results of this study, the authors would like to recommend the following:

First, considering that the elderly desire robots that can represent someone they can live with or family members, technologies should adopt more accurate language, methods of talking, and responses that allow the elderly to emotionally interact with them. To achieve this, robotic technology services mounted with algorithms that accumulate, learn, and show tailored reactions to relevant data (such as older person’s individual expressions), should be developed.

Second, robots should be able to recognize abnormal physical reactions in the elderly at an early stage and notify hospitals or make appropriate decisions (such as diagnosis and treatment or emergency response), especially for individuals living alone.

This study was conducted with a limited number of subjects, all of whom were community-dwelling elderly, their perceptions were based partly on their imagination and expectations regarding robotic technologies; they did not directly use robots or CAS. Therefore, it is difficult to say that this study’s results represent the perceptions of the general elderly population. In this regard, it is necessary to continue research on how the community-dwelling elderly, especially those with various perspectives, health conditions, careers, and financial conditions, perceive robot-related CAS technologies.

For the survey questionnaire, its construct validity and test-retest reliability were not assessed, but experts in the field of ICT and gerontology have verified its face validity and internal reliability. Additional studies need to be conducted to further explore the validity and reliability of this brief and easy-to-use questionnaire not only for older adults but also in other groups.

## Data Availability

The data that support the findings of this study are available from the corresponding author on request.

## Abbreviations

CAS: Connected Active Space
ICT: Information Communication Technology
KIST: Korea Institute of Science and Technology
SAR: Socially Assistive Robots
VAS: Visual Analogue Scale
